# Plantation performance of chestnut hybrids and progenitors on reclaimed Appalachian surface mines

**DOI:** 10.1007/s11056-018-9643-7

**Published:** 2018-04-12

**Authors:** Jeff G. Skousen, Kara Dallaire, Steffany Scagline-Mellor, Alexis Monteleone, Lindsay Wilson-Kokes, Jessica Joyce, Calene Thomas, Travis Keene, Curtis DeLong, Thomas Cook, Douglass F. Jacobs

**Affiliations:** 1 West Virginia University, Morgantown, WV USA; 2 AMEX Inc., Edmonton, AB Canada; 3 USDA Forest Service, Elkins, WV USA; 4 Thomas Jefferson Soil and Water Cons. District, Charlottesville, VA USA; 5 Civil and Environmental Consultants, Knoxville, TN USA; 6 EPA, Washington, DC USA; 7 USGS, Boise, ID USA; 8 Dow Chemical, Harrisburg, PA USA; 9 Ohio EPA, Bowling Green, OH USA; 10 Navigator Environmental Services, Summersville, OH USA; 11 Purdue University, West Lafayette, IN USA

**Keywords:** Forestry reclamation approach, Mine soils, Post-mining land use, Reclamation, Reforestation, Revegetation

## Abstract

Reclamation of surface mined sites to forests is a preferred post-mining land use option, but performance of planted trees on such sites is variable. American chestnut (*Castanea dentata* (Marsh.) Borkh.) is a threatened forest tree in the eastern USA that may become an important species option for mine reclamation. Chestnut restoration using backcross hybrids that incorporate blight resistance may be targeted to the Appalachian coal mining region, which corresponds closely with the species’ native range. Thus, it is important to understand how chestnut hybrids perform relative to progenitors on reclamation sites to develop restoration prescriptions. Seeds of parents and three backcross generations of chestnut (100% American, 100% Chinese, and BC_1_F_3_, BC_2_F_3_, and BC_3_F_2_ hybrids) were planted into mine soils in West Virginia, USA with shelter treatments. Survival for all stock types was 44% after 8 years (American 39%, Chinese 77%, BC_1_F_3_ 40%, BC_2_F_3_ 28%, and BC_3_F_2_ 35%). Height for all stock types was 33 cm after 8 years (American 28 cm, Chinese 67 cm, BC_1_F_3_ 30 cm, BC_2_F_3_ 21 cm, and BC_3_F_2_ 20 cm). At another site a year later, seedlings of the chestnut stock types were planted into brown (pH 4.6) or gray sandstone (pH 6.3) mine soils and seedling survival across all stock types was 58% after 7 years. Chinese had the highest survival at 82%, while the others ranged from 38 to 66%. Height was 63 cm for all stock types after 7 years. More advanced backcross hybrids (BC_2_F_3_ and BC_3_F_2_) had the lowest vigor ratings at both sites after 7–8 years. Our results indicate that surface mines in Appalachia may provide a land base for planting blight-resistant chestnuts, although Chinese chestnut outperformed American chestnut and later generation backcross hybrids. As blight-resistant chestnuts establish and spread after planting, chestnut trees may become a component of the forest canopy again and possibly occupy its former niche, but their spread may alter future forest stand dynamics.

## Introduction

Surface coal mining has disrupted about 2.5 million ha of eastern USA forests since 1930 (Paone et al. 1978; Plass 2000). With the passage of a national surface mining law in the 1970s, most surface mined land in Appalachia was reclaimed to pasture and hay land or wildlife habitat post-mining land uses (Plass 2000; Angel et al. 2005) rather than returning the land to its original forest pre-mining land use. But since the early 2000s, reclamation to forests has become a more preferred post-mining land use option than pasture or hay land in Appalachia (Angel et al. 2009). The Forestry Reclamation Approach (FRA) provides operators with reclamation practices to help restore forests on mined lands. The FRA recommends the following steps: (i) Create a suitable rooting medium for good tree growth that is no less than 1.3 m deep and comprised of topsoil, weathered sandstone, and/or the best available material; (ii) Loosely grade the topsoil or topsoil substitutes established in step one to create a non-compacted growth medium; (iii) Use ground covers that are compatible with growing trees; (iv) Plant two types of trees—(1) early succession species for wildlife and soil stability, and (2) commercially valuable crop trees; (v) Use proper tree planting techniques (Burger et al. 2005). Recent research has shown successful establishment of native hardwood trees when applying all five steps of the FRA (Angel et al. 2008; Wilson-Kokes et al. 2013a, b).

American chestnut (*Castanea dentata* (Marsh.) Borkh.) is a threatened native forest tree in the eastern USA that may become an important species option for mine reclamation (Jacobs 2004, 2007). American chestnut once occupied up to 25% of standing volume in eastern North American forests prior to 1900 (Russell 1987). Trees produced huge volumes of timber because they grew straight, fast, and tall; and were also valued for their consistent annual nut production (Wang et al. 2013). The Appalachian coal mining region corresponds closely with the native range of the American chestnut (Jacobs 2004; Fields-Johnson et al. 2010; Dalgleish et al. 2016).

Chestnut blight, caused by the fungus *Cryphonectria parasitica* (Murr.) Barr, was accidentally introduced into the USA in the early 1900s and destroyed chestnut throughout its range by 1950 (Anagnostakis 1995; Brewer 1995; Jacobs et al. 2013). The USDA Forest Service began a breeding program to develop blight-resistant chestnuts in 1909, which continued until 1960 (Jacobs et al. 2017). Hybrid chestnut plantings were conducted in Connecticut and Virginia during the 1970s and 1980s. The American Chestnut Foundation (TACF), formed in 1983, continued the restoration program to breed American chestnut with blight-resistant Chinese chestnut (*Castanea mollissima* Blume) (Burnham et al. 1986). Hybrids were selected for repeated backcrossing to American chestnut to produce a BC_3_F_3_ chestnut (94% American chestnut and 6% Chinese chestnut) (Hebard 2005). Through careful selection for blight resistance and desirable phenotype, these backcross hybrids incorporated Chinese chestnut’s blight resistance while retaining the timber and nut-producing characteristics of American chestnut (Hebard 2005; Steiner et al. 2017). Although repeated breeding and additional backcross generations will be necessary (Steiner et al. 2017), initiation of chestnut restoration appears imminent and researchers have examined regeneration of pure and backcross American chestnut hybrids across varying environments (see citations in Jacobs 2007; Jacobs et al. 2013; Wang et al. 2013).

A partnership established by TACF and the Appalachian Regional Reforestation Initiative (ARRI) uses reclaimed surface mines in Appalachia to facilitate re-introducing American chestnut with the FRA (OSMRE 2015). American chestnut is a fast-growing tree species with the ability to persist in shaded environments, variable soil conditions, and well-drained, dry upland sites (Latham 1992; Jacobs 2007); yet it responds favorably to increases in sunlight, soil nutrients and moisture (Wang et al. 2006). The original range of American chestnut had average annual precipitation varying from 90 to 150 cm and average annual temperatures from 5 to 15 °C (Fields-Johnson et al. 2010; Wang et al. 2013). Disturbance favors American chestnut over many co-occurring tree species because it sprouts prolifically (Wang et al. 2013). This insight suggests that chestnut may perform relatively well and persist on mine reclamation sites. Chestnut plantings have demonstrated this success in many of the Appalachian states (Kentucky—Barton et al. 2015; Virginia—Fields-Johnson et al. 2012; West Virginia—Skousen et al. 2013; and Ohio—Bauman et al. 2008). Chestnut plantings on reclaimed lands may also provide islands for the spread of blight-resistant chestnut seeds into surrounding forests (Jacobs 2007) and may help to re-establish chestnuts to their native range.

In developing silvicultural prescriptions to restore threatened species following hybrid backcross breeding, it is important to understand how hybrids perform relative to progenitors under varying environmental conditions (Crystal and Jacobs 2014; Crystal et al. 2016). Chinese chestnut appears to have similar silvics to American chestnut, and Chinese chestnuts have been found naturalized in eastern USA forests in analogous environments to those inhabited by American chestnut (Miller et al. 2014). Where these two species have been planted in studies, survival and growth tends to be quite similar (Barton et al. 2015). The few studies that have planted chestnut hybrids and progenitors in analogous environments have shown that the hybrids performed similarly to the parents with a tendency toward American chestnut survival and growth (Barton et al. 2015; Bauman et al. 2014; Fields-Johnson et al. 2010; Pinchot et al. 2014; Skousen et al. 2013), yet these studies have generally evaluated only the early phases of regeneration establishment.

The objectives of this research were to determine the plantation development of two chestnut parental species and three backcross breeding generations, hereafter called ‘stock types,’ (American, Chinese, BC_1_F_3_, BC_2_F_3_, and BC_3_F_2_; see Hebard (2005) for descriptions of backcross chestnuts) in mine soils. In Study 1, seeds were planted with and without tree shelters in a mixed brown/gray mine soil. In Study 2, seedling establishment and growth of these five chestnut stock types were evaluated in two distinct mine soils: a loosely-dumped brown sandstone material and a compacted gray sandstone material. The first 4 years of survival and growth for Study 1, and 3-year survival and growth for Study 2 were published previously (Skousen et al. 2013). In Study 1, first year establishment from seeds averaged 81%. After 4 years, survival without shelters had declined for all chestnut stock types except Chinese (80%): American 40%, BC_1_F_3_ 70%, BC_2_F_3_ 40%, and BC_3_F_2_ 55%. Survival with shelters was only slightly better after 4 years (mean 60% with shelters and 57% without). Height growth was not different among stock types and mean height after 4 years was 43 cm without shelters and 56 cm with shelters. In Study 2, very few seeds germinated. Transplanted chestnut seedling survival, however, ranged from 48 to 100% survival across stock types. After 3 years, seedling survival averaged 85% in brown mine soils and 80% in gray mine soils, but significant differences were found with stock types. Survival was significantly higher with American, Chinese, and BC_1_F_3_ stock types (75%) than BC_2_F_3_ and BC_3_F_2_ (60%). Average height (cm) after 3 years averaged 90 cm on brown and 62 cm on gray. Here, we report the results after 8 years for Study 1 and 7 years for Study 2.

## Materials and methods

### Study 1: seeds

Study 1 was established in 2008 at the Glory surface mine, west of Madison in Boone County, West Virginia (37°57′45.48″N, 81°45′01.04″W). Precipitation averages 112 cm and is evenly distributed throughout the year (Wolf 1994). The average annual temperature during the growing season is 20 °C. The planting site of about 2 ha was prepared by end dumping a mixture of brown and gray sandstone materials and grading the land surface with a bulldozer. The experimental design was a randomized block design with eight blocks, each containing five plots. Ten seeds of each of the five chestnut stock types were planted into randomly-selected plots at 2.4-m spacing (see Skousen et al. (2013) for more details). All chestnut seeds (and seedlings for Study 2) were provided by TACF from Meadowview, VA. Study 1 was originally incorporated a peat/no peat treatment and a shelter/no shelter treatment (45-cm-tall, blue, plastic tree shelter (Blue-X^®^) with perforations). Survival and growth results after 4 years showed no significant difference between peat treatments and only slight differences between shelter treatments. Here we compare survival and growth of the five chestnut stock types in Study 1 disregarding peat treatments but keeping the shelter treatments. Survival was determined for each planted seed and if present its height was measured. A vigor rating from 1 to 5 was also assigned to each live tree: 1—> 75% leaves discolored, extensive dieback; 2—50–75% leaves discolored, dieback present: 3—25–50% leaves discolored, dieback present; 4—25–50% leaves discolored, no dieback present; and 5—< 25% leaves discolored, no dieback present. Trees were measured during August of the years 2008–2011 and in 2015, but only data from 2011 and 2015 are shown here.

Statistical analyses were performed using SAS 9.1 software (SAS Institute 2005). For each year, data for tree survival and height for each shelter treatment were analyzed by ANOVA (Proc GLM) with stock type as the main effect variable (*p* < 0.05). Means were separated with Tukey’s honest significant difference tests (SAS Institute 2005) using an alpha level of 0.05 as significant.

### Study 2: seedlings

Study 2 was established in 2009 at the Nicholas surface mine about 15 km west of Summersville, WV (38°19′45.22″N, 80°58′31.25″W). Two mine soil types (brown or gray) were available for planting at this site. The brown sandstone materials came from the surface 10-m of material, which was end dumped by trucks with no smoothing of the piles. The gray sandstone topsoil substitute material came from the overburden at a depth of 40 m and was composed of unweathered, coarse-textured materials, which was dumped and smoothed with bulldozers. Precipitation at the site averages 118 cm with an average temperature of 18 °C (Carpenter 1992). This study used a randomized block design composed of five blocks on each substrate and five plots in each block. Originally, each block had 10 plots, where each plot was planted with either five seeds or five seedlings of each stock type but no seeds germinated and we found no trees establishing from seeds after 1 year or after 4 years (Skousen et al. 2013). Therefore, Study 2 in this paper reports only the results of the seedling planting of the five chestnut stock types in the two substrates. The seedling planting procedure involved digging holes large enough for the roots of the seedlings. Survival, height and vigor of each live chestnut seedling were determined in August 2009–2011 and in 2015, but data from only 2011 and 2015 are reported here.

Statistical analyses were performed using SAS 9.1 software (SAS Institute 2005). For each individual year, data for tree survival and height of tree seedlings in each substrate were analyzed with ANOVA (Proc GLM) with stock type as the main effect (*p* < 0.05). Means were separated with Tukey’s honest significant difference tests (SAS Institute 2005) using an alpha level of 0.05 as significant.

### Soil sampling

For the three mine soil types (Study 1—mixed mine soil areas with and without shelters, Study 2—brown and gray), soil samples were extracted at five random locations to a depth of 15 cm to evaluate chemical properties. Samples were analyzed for pH (1:1 soil:water) with a Beckman 43 pH meter and elemental content by the West Virginia University Soil Testing Laboratory with a Mehlich 1 extract, which is composed of approximately 0.05 *N* HCl and 0.025 *N* H_2_SO_4_. The leachate from the extraction was analyzed with a Perkin Elmer Plasma 400 emission spectrometer for H, Al, P, K, Ca, and Mg. Cation exchange capacity was calculated by summing the above elements and base saturation was calculated as the sum of base cations divided by total cations. Statistical analyses were performed for each parameter within each study with ANOVA (Proc GLM) (*p* < 0.05) and means were separated with Tukey’s honest significant difference tests (SAS, 2005) using an alpha level of 0.05.

## Results and discussion

### Study 1: Seeds

Soil analysis revealed a pH range of 5.6–6.3 for mine soils in Study 1 (Table 1). There were no differences in the mine soil chemical properties across the areas with and without shelters. These values were found to be typical for a mixture of brown and gray sandstone mine soil material (Haering et al. 2004; Angel et al. 2008; Showalter et al. 2007; Emerson et al. 2009) and were generally suitable for chestnut tree growth (Wang et al. 2013).

**Table 1:** Chemical properties of mine soils for Studies 1 and 2 where five chestnut stock types were planted on surface mines in West Virginia

Property	Study 1		Study 2	
	Without shelter	With shelter	Brown	Gray
pH	5.6 (0.2)	6.3 (0.3)	4.6 (0.5)b	6.3 (0.4)a
EC (ds m^−3^)	0.05 (0.01)	0.05 (0.01)	0.02 (0.02)	0.06 (0.02)
P (mg kg^−1^)	45 (17)	52 (13)	9 (6)b	21 (10)a
K (cmol^+^ kg^−1^)	0.22 (0.14)	0.34 (0.20)	0.26 (0.11)	0.10 (0.09)
Ca (cmol^+^ kg^−1^)	2.6 (0.9)	2.4 (1.2)	2.3 (1.3)b	6.1 (0.8)a
Mg (cmol^+^ kg^−1^)	2.3 (0.3)	2.9 (0.4)	1.6 (0.5)b	2.8 (0.6)a
CEC (cmol^+^ kg^−1^)	12 (3)	13 (2)	12 (2)	11 (4)
BS (%)	48 (16)	62 (12)	37 (9)b	68 (11)a

Standard errors are in parenthesis

*Values for each study within years with different letters are significantly different at *p* < 0.05. If no letters, the values are not significantly different

Survival for all stock types decreased from a mean of 58% after 4 years to a mean of 44% after 8 years (trees without shelters declined from 57 to 38%, and with shelters from 60 to 50%, Table 2). Height declined from a mean of 50 cm to 33 cm (without shelters, 43–25 cm, and with shelters, 56–41 cm).

**Table 2:** Mean seedling survival and height of five chestnut stock types in Study 1 (planted seeds) with and without shelters 4 and 8 years after planting

Stock type	Without shelters		With shelters	
	2011^#^	2015	2011^#^	2015
**Survival (%)**				
American	40b*	33b	50b	45b
Chinese	80a	63a	90a	90a
BC_1_F_3_	70a	40ab	60b	40b
BC_2_F_3_	40b	23b	50b	34b
BC_3_F_2_	55ab	30b	50b	40b
Ave.	57	38	60	50
SE	14	10	16	20
**Height (cm)**				
American	40	20b	59	36b
Chinese	41	50a	61	84a
BC_1_F_3_	45	23b	51	38b
BC_2_F_3_	47	15b	59	28b
BC_3_F_2_	45	19b	51	21b
Ave.	44	25	56	41
SE	8	12	7	9

Standard errors are listed for each shelter treatment

^#^2011 survival and height data were published in Skousen et al. (2013)

*Values within columns for survival or height with different letters are significantly different at *p* < 0.05

For individual stock types, American survival without shelters declined from a mean of 40% after 4 years to 33% after 8 years. The others declined more: Chinese from 80 to 63%, BC_1_F_3_ from 70 to 40%, BC_2_F_3_ from 40 to 23%, and BC_3_F_2_ from 55 to 30% (Table 2). With shelters, survival for American went from 50% after 4 years to 45% after 8 years; Chinese stayed constant at 90%, BC_1_F_3_ from 60 to 40%, BC_2_F_3_ from 50 to 34%, and BC_3_F_2_ from 50 to 40%.

Survival of the different stock types in Study 1 without shelters after 4 years showed that Chinese and the single backcross hybrid (BC_1_F_3_) performed similar to each other, while second and third backcross hybrids (BC_2_F_3_ and BC_3_F_2_) had survival similar to the American parent.

The seedlings in this study were only protected with shelters for the first 1½ years, and it was thought that this limited protection period would eventually result in no difference in survival and growth. McCarthy et al. (2010) and Barton et al. (2015) also found that survival of chestnut seedlings was higher with tree shelters. Tree shelters protect seeds and seedlings from predators and may improve growing conditions (Ponder 2003), but they may also induce high temperatures within the shelter (Bergez and Dupraz 1997; Oliet and Jacobs 2007). Although tree shelters had been removed 1½ years after planting, there was still a benefit on survival and growth after 8 years (38% survival for seedlings without shelters and 50% survival with shelters), which supports past studies showing sustained improvement of seedling performance even several years after shelter removal (Jacobs 2011).

Seedling heights for all stock types declined 10–30 cm, except for Chinese, which grew 10–20 cm (Table 2). After 8 years for both unsheltered and sheltered trees, Chinese chestnut trees were nearly double the height and significantly taller than the other four stock types after 8 years (Table 2). Height of the hybrids were similar to American height after 8 years, and all were significantly lower than that of Chinese. The reason(s) for declines in average height for all stock types except Chinese is not clear. However, the average vigor ratings provided evidence of the poor growth (Table 3). The vigor ratings after 4 years varied from 2.8 to 3.4. But after 8 years, Chinese had an average vigor at 2.8, while all others varied between 2.4 and 2.0 (Table 3). The average ratings of 2–3 indicated that these trees had 25–75% discolored leaves with dieback present. Signs of blight and sunscald were not evident on the trunks of these chestnut seedlings (although we saw signs of trunk damage on some trees), and herbivory and browsing were not readily apparent, yet the trees were growing poorly.

**Table 3:** Mean seedling vigor of five chestnut stock types (planted seeds) 4 and 8 years after planting

Stock type	Vigor	
	2011	2015
American	3.2	2.5b*
Chinese	3.4	2.8a
BC_1_F_3_	3.2	2.4b
BC_2_F_3_	3.2	2.0c
BC_3_F_2_	2.8	2.0c
Ave.	3.2	2.3
SE	0.5	0.2

The seedling vigor values for Study 1 with and without shelters were averaged together. See Methods for rating descriptions. Standard error is for all stock types

*Values within columns with different letters are significantly different at *p* < 0.05. Values with no letters signify no significant difference

Height of planted red oak seedlings was between 100 and 150 cm and white oak was between 150 and 200 cm on brown sandstone substrates in WV after 8 years (Dallaire et al. 2015), which is more than double the height of our chestnut trees after 8 years. In Indiana mine soils, McCarthy et al. (2008) found that seedling height of backcross stock types (BC_1_ and BC_2_) was 60 cm versus 35 cm for American after 1 year in Indiana mine soils, but after 5 years, these chestnut seedlings had increased to 150 cm (Bauman et al. 2014).

### Study 2: Seedlings

Soil chemical properties were significantly different for most parameters between the brown and gray mine soils in Study 2 (Table 1). Soil pH in brown was much lower at 4.6 compared to pH 6.3 for gray mine soils. The pH of gray sandstone mine soil substitutes in West Virginia is generally higher, sometimes nearly pH 8.0 (Emerson et al., 2009). The higher P concentrations in gray versus brown mine soils have also been documented in studies of West Virginia mine soils (Emerson et al., 2009; Thomas and Skousen, 2011), but this greater P in these soils may not be plant available as shown by a leaching study (Skousen and Emerson, 2010). Significantly greater quantities of Ca and Mg were found in gray versus brown mine soils, which caused much higher base saturation values. Chestnut trees prefer soils with pH from 5 to 6, but they grow on a wide range of soil pH and under a wide range of soil nutrient concentrations (French et al. 2007; McCarthy et al. 2008; Wang et al. 2013).

In brown mine soils, survival for all stock types declined from a mean of 71% after 3 years to 51% after 7 years, while gray decreased less so from 73 to 64% during the same time (Table 4). In brown, all stock types decreased in survival (e.g., American went from 80 to 68% and BC_3_F_2_ from 64 to 28%. In gray, three stock types did not decline: Chinese, B_2_F_3_ and BC_3_F_2_. Gilland and McCarthy (2012) found that chestnut seedling survival increased where some herbaceous vegetation cover was present and where compaction was reduced.

**Table 4:** Mean seedling survival and height for five chestnut stock types in Study 2 (planted seedlings) in brown and gray mine soils 3 and 7 years after planting

Stock type	Brown		Gray	
	2011^#^	2015	2011^#^	2015
**Survival (%)**				
American	80a	68a*	80a	56b
Chinese	84a	68a	100a	96a
BC_1_F_3_	72ab	56ab	92a	76ab
BC_2_F_3_	56b	36b	44b	44b
BC_3_F_2_	64b	28b	48b	48b
Ave.	71	51	73	64
SE	9	15	21	13
**Height (cm)**				
American	92ab	110a	71a	56b
Chinese	112a	106a	79a	80a
BC_1_F_3_	85ab	86ab	52b	64b
BC_2_F_3_	68b	26c	53b	24c
BC_3_F_2_	82ab	46bc	39c	33c
Ave.	88	75	59	51
SE	11	18	10	7

Standard errors are listed for each substrate treatment

^#^2011 survival and height data were published in Skousen et al. (2013)

*Values within columns (year) for survival or height with different letters are significantly different at *p* < 0.05

Heights of seedlings in brown mine soils did not decline for American, Chinese, and BC_1_F_3_ between years 3 and 7, but the other two hybrids declined between 20 and 30 cm from year 3 to 7 (Table 4). It was encouraging that three of the stock types increased in height on the brown mine soils, whereas only Chinese increased in height in Study 1. On gray mine soils, height increased for Chinese and BC_1_F_3_, but declined for the others between years 3 and 7 (Table 4). Bauman et al. (2014) reported that chestnut seedlings were 150–180 cm tall after 5 years in Indiana mine soils, much taller than the chestnuts growing in this West Virginia study.

The poor survival of BC_2_F_3_ and BC_3_F_2_ to around 32% in brown and 46% in gray (Table 4) was troubling considering the other stock types had higher survival (56 to 96% after 7 years). Poor height growth was also evident for these two hybrids. The difference in mean survival between brown (51%) and gray (64%) was due to the higher Chinese survival on gray in 2015 (68 vs. 96%). The high Chinese chestnut survival in gray mine soils is puzzling as gray mine soils are considered poor substrates for hardwood tree survival and growth (Skousen et al. 2011a, b). It is possible that Chinese chestnut may be better adapted to higher pH soils and soils high in Ca compared to American chestnut. As supporting evidence, the BC_1_F_3_ hybrid survived and grew better on the gray mine soils than the BC_2_F_3_ and BC_3_F_2_ hybrids and American during the 7-year study. But survival and particularly growth of other hardwood trees like oaks, poplars, and maples is generally much better in brown versus gray mine soils (Wilson-Kokes et al. 2013a, b; Zipper et al. 2013).

Vigor of seedlings averaged 3.1 after 3 years in brown mine soils, and ranged from 3.7 for Chinese to 2.6 for BC_2_F_3_. All had higher vigor ratings after 7 years except for BC_3_F_2_. Seedlings growing in gray mine soils were similar to seedling vigor in brown with an average of 3.6, and declined to an average vigor rating of 2.9 (Table 5). Assuming the planted seedlings were similar in size and health when planted in both substrates, the slightly lower vigor ratings and the lower mean heights on gray after 7 years suggests the poorer growing conditions.

**Table 5:** Mean seedling vigor of five chestnut stock types for Study 2 (planted seedlings) in brown and gray mine soils 3 and 7 years after planting

Stock type	Vigor			
	Brown		Gray	
	2011	2015	2011	2015
American	3.2ab*	4.0a	3.5	3.3
Chinese	3.7a	4.0a	3.8	3.0
BC_1_F_3_	2.8bc	3.9a	3.5	2.8
BC_2_F_3_	2.6c	3.4ab	3.4	2.7
BC_3_F_2_	3.3ab	2.8b	3.7	2.7
Ave.	3.1	3.6	3.6	2.9
SE	0.5	0.4	0.4	0.4

See Methods for rating descriptions. Standard errors are listed for each substrate type

*Values within columns (year) for vigor on each substrate with different letters are significantly different at *p* < 0.05. Values with no letters mean no significant difference

This study showed that Chinese chestnut seeds and seedlings survived and grew better than the other stock types over 7–8 years in these three mine soil types, which were brown alone and gray alone in Study 2, and a mixture of the two in Study 1. The first backcross (BC_1_F_3_) tended to survive and grow at levels in between the pure American and Chinese chestnut stock types, while the other two hybrids (BC_2_F_3_ and BC_3_F_2_) generally performed at the same level or worse than American. No hybrid vigor was demonstrated in this study. Because a predominantly American chestnut backcross hybrid (i.e., BC_3_F_3_) is expected to be used for American chestnut restoration, our results suggest that such progeny may perform more poorly than hybrids that are closer to the Chinese progenitor on mine sites characteristic of those tested here.

Chinese and hybrid chestnuts, which are less affected by the blight, could spread into the forests of eastern North America following restoration efforts, potentially releasing foreign tree species with invasive qualities to North American forests (Jacobs 2007). Examples of disastrous releases of introduced species have occurred where natural controls were lacking and the species became invasive (e.g., tree of heaven (*Ailanthus altissima* (Mill.) Swingle), multiflora rose (*Rosa multiflora* Thunb.), and autumn olive (*Elaeagnus umbellata* Thunb.)). Miller et al. (2014) reported the establishment of Chinese chestnut in the USA yet indicated that this species had not developed invasive characteristics. Based on their research with naturalized Chinese chestnut, they suggested that introduced species should be evaluated not just on their invasive characteristics but also on their potential positive contributions to ecosystems. Indeed, ecological risks accompany the introduction of alien species but these risks must be balanced with current and future ecosystem modifications related to climate change, forest pathogens and pests, competition from other species, and ecosystem dynamics (Jacobs et al. 2015). Chinese chestnut as well as other chestnut hybrids that do not succumb to chestnut blight (Jacobs 2007; Wang et al. 2013) can become important sustainable tree species that supply some of the favorable features of American chestnut to forest ecosystems.

This study and others demonstrate the potential of these chestnut stock types to survive and persist, and thereby spread gradually into eastern North American forests by restoration efforts on reclaimed surface mines in Appalachia (Barton et al. 2015; Bauman et al. 2014; Fields-Johnson et al. 2010, and herein). The introduction of Chinese chestnut and chestnut hybrids into eastern North American forests may alter tree species competition and forest ecosystem dynamics, shift the potential for exotic insects and pathogens to affect native species, and invade into areas outside the original native range of chestnuts (Jacobs 2007). Conversely, this re-introduction holds promise to serve as a great success story for restoring a vanquished species to its native range and to potentially occupy its former niche.
